# Determination of CSF GFAP, CCN5, and vWF Levels Enhances the Diagnostic Accuracy of Clinically Defined MS From Non-MS Patients With CSF Oligoclonal Bands

**DOI:** 10.3389/fimmu.2021.811351

**Published:** 2022-02-04

**Authors:** Fay Probert, Tianrong Yeo, Yifan Zhou, Megan Sealey, Siddharth Arora, Jacqueline Palace, Timothy D. W. Claridge, Rainer Hillenbrand, Johanna Oechtering, Jens Kuhle, David Leppert, Daniel C. Anthony

**Affiliations:** ^1^ Department of Chemistry, University of Oxford, Oxford, United Kingdom; ^2^ Department of Pharmacology, University of Oxford, Oxford, United Kingdom; ^3^ Department of Neurology, National Neuroscience Institute, Singapore, Singapore; ^4^ Duke-National University of Singapore (NUS) Medical School, Singapore, Singapore; ^5^ Translational Stem Cell Biology Branch, National Institutes of Health, Bethesda, MD, United States; ^6^ Wellcome Medical Research Council (MRC) Trust Stem Cell Institute, University of Cambridge, Cambridge, United Kingdom; ^7^ Department of Mathematics, University of Oxford, Oxford, United Kingdom; ^8^ Nuffield Department of Clinical Neurosciences, John Radcliffe Hospital, University of Oxford, Oxford, United Kingdom; ^9^ Biomarker Development, Novartis Pharma AG, Basel, Switzerland; ^10^ Neurologic Clinic and Policlinic, Multiple Sclerosis (MS) Center and Research Center for Clinical Neuroimmunology and Neuroscience Basel (RC2NB), Departments of Clinical Research and Biomedicine, University Hospital Basel, University of Basel, Basel, Switzerland

**Keywords:** diagnosis, metabolomics (OMICS), multiple sclerosis (MS), proteomics, oligoclonal band

## Abstract

**Background:**

Inclusion of cerebrospinal fluid (CSF) oligoclonal IgG bands (OCGB) in the revised McDonald criteria increases the sensitivity of diagnosis when dissemination in time (DIT) cannot be proven. While OCGB negative patients are unlikely to develop clinically definite (CD) MS, OCGB positivity may lead to an erroneous diagnosis in conditions that present similarly, such as neuromyelitis optica spectrum disorders (NMOSD) or neurosarcoidosis.

**Objective:**

To identify specific, OCGB-complementary, biomarkers to improve diagnostic accuracy in OCGB positive patients.

**Methods:**

We analysed the CSF metabolome and proteome of CDMS (n=41) and confirmed non-MS patients (n=64) comprising a range of CNS conditions routinely encountered in neurology clinics.

**Results:**

OCGB discriminated between CDMS and non-MS with high sensitivity (85%), but low specificity (67%), as previously described. Machine learning methods revealed CCN5 levels provide greater accuracy, sensitivity, and specificity than OCGB (79%, +5%; 90%, +5%; and 72%, +5% respectively) while glial fibrillary acidic protein (GFAP) identified CDMS with 100% specificity (+33%). A multiomics approach improved accuracy further to 90% (+16%).

**Conclusion:**

The measurement of a few additional CSF biomarkers could be used to complement OCGB and improve the specificity of MS diagnosis when clinical and radiological evidence of DIT is absent.

## Introduction

There remains no single pathognomonic clinical feature or diagnostic test for MS. Diagnosis relies on the integration of clinical, imaging, and laboratory findings within the framework of the McDonald criteria. The present criteria recognise the need to demonstrate clinical and/or radiological dissemination in space (DIS) and time (DIT) and to exclude alternative diagnoses. In 2017, revisions to the McDonald criteria introduced the presence of cerebrospinal fluid (CSF)-specific oligoclonal IgG bands (OCGB) as a proxy of DIT to establish MS diagnosis in patients with a typical clinically isolated syndrome (CIS) and with only evidence of DIS. The OCGB appear to arise from CSF-persistent, clonally related B cell populations, which appears, at least partially, independent of B cell targeted therapy (1). While, these revisions have increased the sensitivity of the McDonald criteria, to facilitate earlier treatment, they have reduced the specificity for clinically definite MS (CDMS) (2).

While CSF OCGB are present in a high proportion of individuals with CDMS (3), they are also detectable in the CSF of individuals with other autoimmune and infectious diseases of the CNS including syndromes with clinical and radiographic overlap with MS (4, 5), and in other non-inflammatory neurological diseases such as migraine (6). Indeed, migraine, fibromyalgia, psychogenic disorders, and neuromyelitis optica spectrum disorders, have all been highlighted as the true cause of illness in patients misdiagnosed with MS (7–9). A recent meta-analysis gave a sensitivity of 84% and a specificity of 54% when using OCGB to predict conversion to CDMS, with a corresponding positive predictive value of 0.64 and a NPV of 0.77 (10). Therefore, when DIT cannot be proven clinically or radiologically, other biomarkers would be useful to improve the specificity of MS diagnosis.

We have previously shown that we can distinguish between individuals with MS and healthy controls with very high accuracy (100%) using NMR-based metabolomics on blood samples (11). However, except for radiologically isolated syndrome (RIS), it is rare that the subject of an investigation for suspected demyelinating disease is healthy on presentation. Thus, the need to distinguish between individuals with MS and the heterogeneous cross-section of non-MS patients encountered in neurology clinics is the normal challenge. A multi-omics approach on samples CSF combined with cross platform multivariate pattern recognition methods affords an opportunity to identify new biomarkers for MS diagnosis.

Here we sought to discover if a CSF-based multivariate diagnostic test combining proteomics, metabolomics, and OCGB could improve the diagnostic accuracy of MS from the heterogeneous mix of neurological diseases encountered in a clinical setting. Using an integrative approach, we looked for diagnostic biomarkers that are independent of OCGB and highly specific for CDMS. Such biomarkers could be added to the 2017 McDonald criteria, alongside highly sensitive OCGB, to improve diagnostic specificity and the positive predictive value in patients where DIT cannot be proven clinically or radiologically. We report a multivariate model which out-performs, not only OCGB status, but all identified metabolite and protein biomarkers when measured in isolation. All models were validated on independent test data using 10-fold cross validation and permutation testing to ensure significance was not a result of the model overfitting the data.

## Materials and Methods

### Study Participants

CSF samples from 41 patients with CDMS (Poser criteria (12)) and 64 patients with non-MS diagnoses (with spinal taps performed as part of their diagnostic investigations) were collected at the Department of Neurology, University Hospital Basel to identify biomarkers specific for CDMS that are independent of OCGB status. Those with a non-MS diagnosis were chosen to represent the heterogenous range of neurological conditions observed in a typical generalist neurology clinic including epilepsy, functional neurological disorders, primary headache syndromes, inflammatory neurological conditions, and infections amongst others (**Table 1**). In summary, the confirmed diagnoses of the non-MS cohort were; primary headache disorder [n=13], functional neurological disorder [n=12], sensory disturbance [n=8], epilepsy [n=5], polyneuropathy [n=5], motor paresis [n=3], neuroinfection [n=3], meningitis [n=2], movement disorder [n=2], myasthenia gravis [n=2], polyradiculitis [n=2], white matter lesions/leukoencephalopathy [n=2], gait disorder [n=1], neuralgic amytrophy [n=1], OCGB+ve normal pressure hydrocephalus [n=1], systemic lupus erythematosus [n=1], visual disturbance [n=1].

**Table 1:** Patient demographics grouped by diagnosis.

	CDMS [n=41]	Non-MS [n=64]	p-value
Female, No. [%]	34 [82.9]	36 [56.3]	0.005
Age at sampling, mean [SD], years	36.4 [9.6]	52.3 [15.6]	<0.001
EDSS, median [IQR], years	2.5 [1.5 - 3.5]	NA	NA
Immune modulating therapies	Fingolimod [n=3], Rebif [n=1], Betaferon [n=2], Avonex [n=3]	NA	NA
Co-morbidities within CDMS cohort/non-MS diagnosis	Migraine [n=5], Hypertension [n=1], inactive herpes simplex [n=1], vitamin B12 deficiency [n=2], vitamin D deficiency [n=2]	Epilepsy [n=5], functional neurological disorder [n=12], gait disorder [n=1], meningitis [n=2], motor paresis [n=3], movement disorder [n=2], myasthenia gravis [n=2], neuralgic amytrophy [n=1], neuroinfection [n=3], OCGB+ve normal pressure hydrocephalus [n=1], polyneuropathy [n=5], polyradiculitis [n=2], primary headache disorder [n=13], sensory disturbance [n=8], systemic lupus erythematosus [n=1], visual disturbance [n=1], white matter lesions/leukoencephalopathy [n=2]	NA
CSF lactate, mean [SD]	1.6 [0.02]	1.3 [0.5]	0.15
CSF glucose, mean [SD]	3.2 [0.3]	3.4 [0.4]	0.07
CSF/plasma glucose ratio, mean [SD]	0.7 [0.2]	0.6 [0.1]	0.04
CSF Leukocyte, mean [SD]	4.9 [5.9]	6.9 [21.4]	0.28
CSF total protein, mean [SD]	383.7 [140.6]	384.7 [140.6]	0.49
CSF/plasma albumin ratio, mean [SD]	4.7 [2.3]	5.6 [1.9]	0.11
OCGB positive, number [%]	35 [85.3]	21 [32.8]	<0.001

P-values from Student’s t-test for continuous variables and Chi-squared test for categorical variables are reported. IQR, interquartile range; SD, standard deviation; CSF, cerebrospinal fluid; OCGB, oligoclonal bands; EDSS, expanded disability status scale.

NA, not applicable.

### Standard Protocol Approvals, Registrations, and Patient Consents

Written informed consent was obtained from all patients according to the Declaration of Helsinki. Ethical approval was obtained by the local ethics committee (University Hospital Basel ethics # 332/06).

### CSF Sample Collection

CSF samples were centrifuged at 400 × g for 10 minutes at room temperature, and the cell-free supernatant stored at -80°C within 2 hours of collection according to a consensus protocol (13). Standard laboratory procedures measured leukocytes [cells/mm^3^] and total protein concentration [mg/dL]. CSF/serum albumin was calculated using concurrent serum samples. Detection of OCGB was by isoelectric focusing on agarose gel and subsequent immunoblotting using IgG-specific antibody staining (14). Patterns two or three were considered OCGB positive (15).

### NMR Spectroscopy and Data Processing for Metabolomics Analysis

100 µL of CSF was diluted with 450 μL of 75 mM sodium phosphate buffer D_2_O (pH 7.4) containing 1 mM maleic acid as an internal reference standard. Samples were centrifuged at 3,000 x g for 5 minutes before transferring to a 5-mm NMR tube. NMR spectra were acquired at 310 K using a 700-MHz Bruker AVIII spectrometer operating at 16.4 T equipped with a ^1^H [^13^C/^15^N] TCI cryoprobe (Department of Chemistry, University of Oxford) and processed as previously described (16).

NMR metabolite measures were converted to absolute concentrations using the internal reference standard (1 mM maleic acid) as previously described (16). To validate the quantification of the metabolites by NMR, the glucose and lactate levels in all CSF samples were measured using a Cobas^®^ 8000 modular analyser (Roche Diagnostics, Switzerland) coupled with the Gluc3 and LAC2 assays, respectively.

### Protein Profiling by SomaScan™

Protein biomarker profiling was performed using SomaScan^®^ (SomaLogic, USA), a multiplexed proteomic tool that measures more than 5000 protein analytes that recognise 4,137 distinct human gene targets (17, 18).

### Statistical Analysis

Multivariate orthogonal partial least squares discriminant analysis (OPLS-DA) was performed in R software (R foundation for statistical computing, Vienna, Austria) (R Development Core Team, 2019) using in-house R scripts and the ropls package (19). All models were validated on independent data using an external 10-fold cross-validation strategy with repetition coupled with permutation testing as previously described (20). Thus, it should be noted that all models are tested on data that was excluded from model building and that the training and test cohorts never overlap. An in-depth description of this analysis approach can be found in our previous publication (21). Variables responsible for the observed class separation are extracted by inspection of the average variable importance (VIP) scores.

Two-sample t-tests or two-way ANOVA were used for continuous variables and Chi-square tests for categorical variables. A multiple comparisons correction (Bonferroni) was applied throughout. Receiver operator curves (ROC), area under the curve (AUC), 95% confidence intervals, optimal thresholds for diagnosis, and p values (relative to a null distribution ROC curve with AUC =0.5) were calculated for each discriminatory variable using the pROC package (22). Hierarchical clustering was performed on the discriminatory proteins to identify clusters similarly expressed and correlated proteins using the ‘pheatmap’ and ‘corrplot’ packages. Joint-pathway hypergeometric enrichment analysis was performed on the discriminatory proteins and metabolites identified by the multivariate analysis (described above) using MetaboAnalyst 5.0 [http://metaboanalyst.ca, last accessed 05/05/21]. Degree centrality was used as the topology measure along with the combined queries integration method.

## Results

### High Sensitivity and Low Specificity of CSF OCGB When Discriminating Between Clinically Definite MS and Other Non-MS Neurological Diseases

CSF samples from 105 patients seen in the neurology clinic were investigated: 41 with a diagnosis of CDMS and 64 with a confirmed non-MS diagnosis. Demographic and clinical chemistry data can be found in **Table 1**.

**Figure 1:**
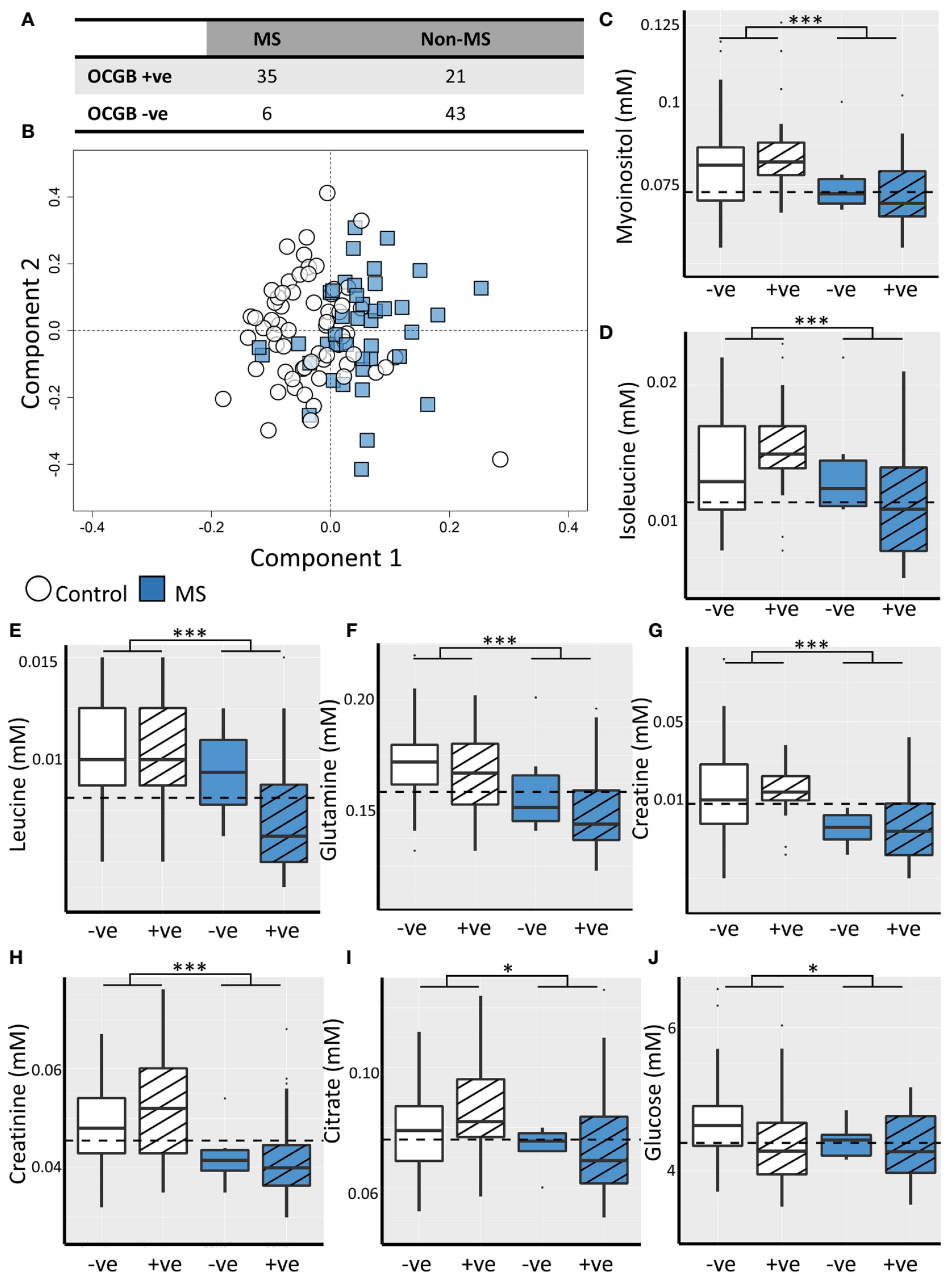
**(A)** Confusion matrix illustrating low specificity of OCGB status for CDMS. **(B)** Representative OPLS-DA scores plot illustrating discrimination between CDMS (blue square, n=41) and non-MS (white circle, n=64) CSF metabolite profiles. Box plots illustrating CSF concentrations of the discriminatory metabolites identified by the OPLS-DA model for OCGB positive (striped) and OCGB negative (solid colour) CDMS (blue) and non-MS (white). The optimal cut-off for each metabolite identified by ROC analysis is represented by a dashed line for **(C)** myo-inositol, **(D)** isoleucine, **(E)** leucine, **(F)** glutamine, **(G)** creatine, **(H)** creatinine, **(I)** citrate, and **(J)** glucose. Bonferroni corrected 2-way ANOVA p-values for disease (MS v. non-MS) effect less than 0.05, and 0.001 are represented by *, and *** respectively. +ve; oligoclonal band positive, -ve; oligoclonal band negative.

Thirty-five of 41 (85%) CDMS patients were positive for OCGB, in the non-MS set this was the case for 21 of 64 patients (32%) (**Figure 1A**). Thus, while the sensitivity of OCGB status alone is high (85%) the specificity in this cohort is only 67% resulting in an overall accuracy and AUC of 74% and 0.74, respectively.

### The CSF Metabolite Profile of CDMS Is Distinct From That of Non-MS Neurological Diseases and Independent of OCGB Positivity

OPLS-DA identified patterns of NMR-detectable metabolites which differ significantly between the CDMS and non-MS cohorts independently of OCGB status, age, and non-MS diagnosis (**Figure S1**) Models identified CDMS and non-MS patients in the test data with accuracy, sensitivity, and specificity of 70 ± 4%, 73 ± 6%, and 70 ± 4% respectively (**Figure 1B**) and permutation testing confirmed that these values are significantly higher than expected from random chance alone (**Figure S2**).

Inspection of the VIP scores illustrated that leucine, isoleucine, glutamine, citrate, creatine, creatinine, glucose, and myo-inositol are significantly decreased in CDMS (**Table 2**). Interestingly, all the metabolites identified discriminate between CDMS and the other neurological conditions independently of OCGB status as confirmed by two-way ANOVA in which no significant interaction between diagnosis (CDMS/non-MS) and OCGB status was observed for any of the biomarkers. Furthermore, no metabolite reached significance when comparing the levels in OCGB+ve samples to OCGB-ve and the OPLS-DA was able to separate MS from non-MS irrespective of OCGB status (**Figure S1B**).

**Table 2:** List of significant CSF metabolites identified by OPLS-DA which drive the discrimination between CDMS and Controls ranked from highest to lowest specificity.

Metabolite	CDMS v non-MS (fold change)	OCGB+ve v. OCGB-ve [p-value]	Interaction [p-value]	AUC	Acc (%)	Sens (%)	Spec (%)	PPV	NPV	TP	FN	TN	FP
Myo-inositol	↓*** (0.87)	ns [0.67]	ns [0.41]	0.74	73	49	89	0.74	0.73	20	21	57	7
Isoleucine	↓*** (0.83)	ns [0.65]	ns [0.08]	0.71	72	49	88	0.71	0.73	20	21	56	8
Leucine	↓*** (0.81)	ns [0.74]	ns [0.18]	0.74	73	61	81	0.68	0.76	25	16	52	12
Glutamine	↓*** (0.89)	ns [0.28]	ns [0.75]	0.76	74	73	75	0.65	0.81	30	11	48	16
*OCGB*	NA	NA	NA	0.74	74	85	67	0.63	0.88	35	6	43	21
Creatine	↓*** (0.89)	ns [0.76]	ns [0.96]	0.75	71	78	67	0.6	0.83	32	9	43	21
Creatinine	↓*** (0.83)	ns [0.25]	ns [0.39]	0.76	71	80	66	0.6	0.84	33	8	42	22
Citrate	↓* (0.90)	ns [0.25]	ns [0.55]	0.61	62	63	61	0.51	0.72	26	15	39	25
Glucose	↓* (0.95)	ns [0.05]	ns [0.5]	0.65	62	71	56	0.51	0.75	29	12	36	28

2-way ANOVA p-values less than 0.001, and 0.05 following Bonferroni correction for multiple comparisons are represented by ***, and * respectively. ns, not significant; ↓, decrease in CDMS relative to non-MS Control. Diagnostic accuracy of OCGB status is included for comparison. AUC, receiver operator curve area under the curve; PPV, positive predictive value; NPV, negative predictive value; OCGB, CSF oligoclonal bands; PPV, positive predictive value; NPV, negative predictive value; TP, true positive; FN, false negative; FP, false positive; TN, true negative.

NA, not applicable.

### CSF Myo-Inositol, Isoleucine, Leucine, and Glutamine Levels Discriminate Between CDMS and Non-MS Neurological Conditions With Greater Specificity Than OCGB Status Alone

The diagnostic utility of each identified metabolite biomarker was investigated using ROC analysis to identify the optimum metabolite concentration cut-off. Metabolite biomarkers ranked by specificity are shown in **Table 2**. Four of the metabolite biomarkers identified (myo-inositol, isoleucine, leucine, and glutamine) have higher specificity than OCGB. While CSF myo-inositol concentrations provide the same AUC as OCGB status (0.74), sensitivity is sacrificed (49%, -36% compared to OCGB status) in favour of specificity (89%, +22%), suggesting that this metabolite is useful for discriminating between CDMS and other neurological conditions which are OCGB+ve. Indeed, myo-inositol levels correctly identified 19 (90%) of the non-MS OCGB+ve cohort as non-MS. **Figures 1C-J** illustrates the improved positive predictive power of myo-inositol, isoleucine, and leucine, particularly in the non-MS OCGB+ve patients.

### The CSF Proteomics Profile of CDMS Is Distinct From That of Non-MS Neurological Conditions and Independent of OCGB Positivity

Next, we investigated the CSF proteomics profiles of the CDMS and non-MS patients. OPLS-DA was able to discriminate between CDMS and other neurological conditions with accuracy, sensitivity, and specificity of 75 ± 4%, 75 ± 4%, and 77 ± 5% respectively independently of OCGB status, age, and non-MS diagnosis (**Figure S3**). This is an improvement on the specificity of OCGB alone which is 67%. Once again, the permutation test confirmed these values are significantly higher than expected from random chance alone (**Figure S4**).

### CCN5, vWF, and GFAP CSF Levels Outperform OCGB Status Alone for the Discrimination of CDMS and Non-MS Neurological Conditions

**Figure 2:**
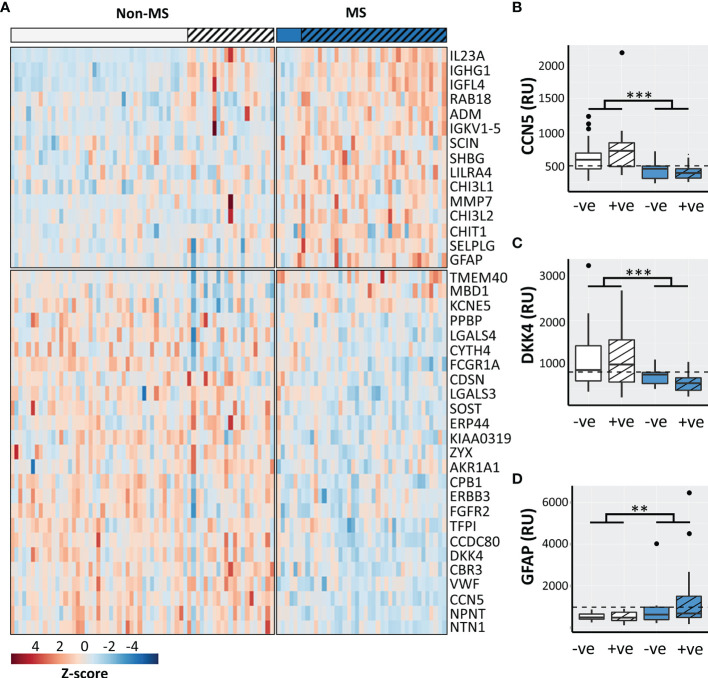
**(A)** Heatmap of the top 40 discriminatory proteins identified. Box plots illustrating CSF concentrations of CDMS OCGB+ve (n=35, blue striped), CDMS OCGB-ve (n=6, solid blue), non-MS OCGB+ve (n=21, white striped), and non-MS OCGB-ve (n=43, solid white) for the two most sensitive **(B, C)** and most specific **(D)** proteins identified. The optimal cut-off for each metabolite identified by ROC analysis is represented by a dashed line. Bonferroni corrected 2-way ANOVA p-values for disease (CDMS v. non-MS) effect less than 0.01 and 0.001 are represented by **, and *** respectively.+ve; oligoclonal band positive, -ve; oligoclonal band negative.

VIP scores identified 40 significantly perturbed proteins driving the separation between CDMS and non-MS patients (**Figure 2A**) of which 13 discriminated CDMS from non-MS with a greater AUC than OCGB (**Table S1**). A significant OCGB effect was present in several IgG-associated proteins (**Table S1**). immunoglobulin G1 (IGHG1), nephronectin (NPNT), and methyl-CpG-binding domain protein 1 (MBD1) were significantly increased in the OCGB positive patients relative to OCGB negative (irrespective of MS diagnosis) while IgG-receptor (FCGR1A) was decreased.

Nine protein biomarkers outperformed OCGB status as predictors of CDMS and their levels are independent of OCGB status. Connective tissue growth factor/Cysteine-rich protein/Nephroblastoma overexpressed-5 (CCN5), coiled-coil domain-containing protein 80 (CDCC80), netrin-1 (NTN1), von Willebrand factor (vWF), dickkopf-related protein 4 (DKK4), sclerostin (SOST), and receptor tyrosine-protein kinase erbB-3 (ERBB3) were significantly decreased in CDMS CSF relative to non-MS while insulin growth factor-like family member 4 (IGL4) and immunoglobulin kappa variable 1-5 (IGKV1-5) were increased (**Table 3**).

**Table 3:** List of the top 14 protein biomarkers identified by OPLS-DA which outperform OCGB for discriminating between CDMS and Controls ranked from highest to lowest AUC.

Uniprot #	Gene	Protein	CDMS v Non-MS (fold change)	OCGB+ve v. OCGB-ve [p-value]	Interaction [p-value]	AUC	Acc (%)	Sens (%)	Spec (%)	PPV (%)	NPV (%)	TP	FN	FP	TN
O76076	CCN5	CCN family member 5 (WISP-2)	↓*** (0.61)	ns [3.18]	ns [4.72]	0.85	79	90	72	67	92	37	4	18	46
Q76M96	CCDC80	Coiled-coil domain-containing protein 80	↓*** (0.79)	ns [30.58]	ns [3.21]	0.83	76	71	80	69	81	29	12	13	51
Q6B9Z1	IGFL4	Insulin growth factor-like family member 4	↑ns (1.57)	ns [0.1]	ns [27.79]	0.81	80	73	84	75	83	30	11	10	54
O95631	NTN1	Netrin-1	↓*** (0.86)	ns [32.8]	ns [23.25]	0.81	73	85	66	61	88	35	6	22	42
P01602	IGKV1-5	Immunoglobulin kappa variable 1-5	↑ns (1.24)	ns [0.91]	ns [39.39]	0.8	81	68	89	80	81	28	13	7	57
P01857	IGHG1	Immunoglobulin heavy constant gamma 1	↑*** (1.79)	*** [<0.001]	ns [14.77]	0.77	77	76	78	69	83	31	10	14	50
Q9NPF7	IL23A	Interleukin-23 subunit alpha	↑ns (1.51)	** [0.006]	ns [14.39]	0.77	78	80	77	69	86	33	8	15	49
Q6UXI9	NPNT	Nephronectin	↓*** (0.82)	ns [30.61]	ns [34.37]	0.77	73	76	72	63	82	31	10	18	46
P04275	VWF	von Willebrand factor (vWF)	↓*** (0.63)	ns [0.45]	ns [6.22]	0.77	70	88	59	58	88	36	5	26	38
Q9UBT3	DKK4	Dickkopf-related protein 4	↓*** (0.59)	ns [37.37]	ns [13.99]	0.76	70	90	56	57	90	37	4	28	36
Q9BS26	ERP44	Endoplasmic reticulum resident protein 44	↓** (0.83)	ns [1.01]	ns [0.17]	0.75	73	83	67	62	86	34	7	21	43
P21860	ERBB3	Receptor tyrosine-protein kinase erbB-3	↓** (0.81)	ns [11.22]	ns [22.35]	0.75	71	78	67	60	83	32	9	21	43
Q9BQB4	SOST	Sclerostin	↓** (0.78)	ns [15.21]	ns [14.8]	0.75	68	80	59	56	83	33	8	26	38
O75828	CBR3	NADPH-dependent carbonyl reductase 3	↓* (0.71)	ns [3.75]	ns [2.89]	0.74	72	66	77	64	78	27	14	15	49
NA	NA	OCGB	NA	NA	NA	0.74	74	85	67	63	88	35	6	43	21

2-way ANOVA p-values less than 0.001, 0.01, and 0.05 following Bonferroni correction for multiple comparisons are represented by ***, **, and * respectively. ns, not significant; ↓, decrease in CDMS CSF relative to Control, ↑, increase in CDMS CSF relative to non-MS control. AUC, receiver operator curve area under the curve; PPV, positive predictive value; NPV, negative predictive value; TP, true positive; FN, false negative; FP, false positive; TN, true negative.

NA, not applicable.

Measurement of the CCN5 concentration resulted in a greater AUC (0.85, + 0.11), accuracy (79%, +5%), sensitivity (90%, +5%), and specificity (72%, +5%) than OCGB status alone (**Table 3**). Glial fibrillary acidic protein (GFAP) (increased in CDMS) results in 100% specificity (+33% relative to OCGB) (**Table S2**). Following threshold optimisation, the top protein VIPs together clearly separate the non-MS OCGB+ve and the CDMS OCGB+ve groups (**Figures 2B**–**D**).

### JAK-STAT Pathways Are Upregulated in CDMS Patients While BCAA Degradation and Tyrosine Metabolism Are Down Regulated

**Figure 3:**
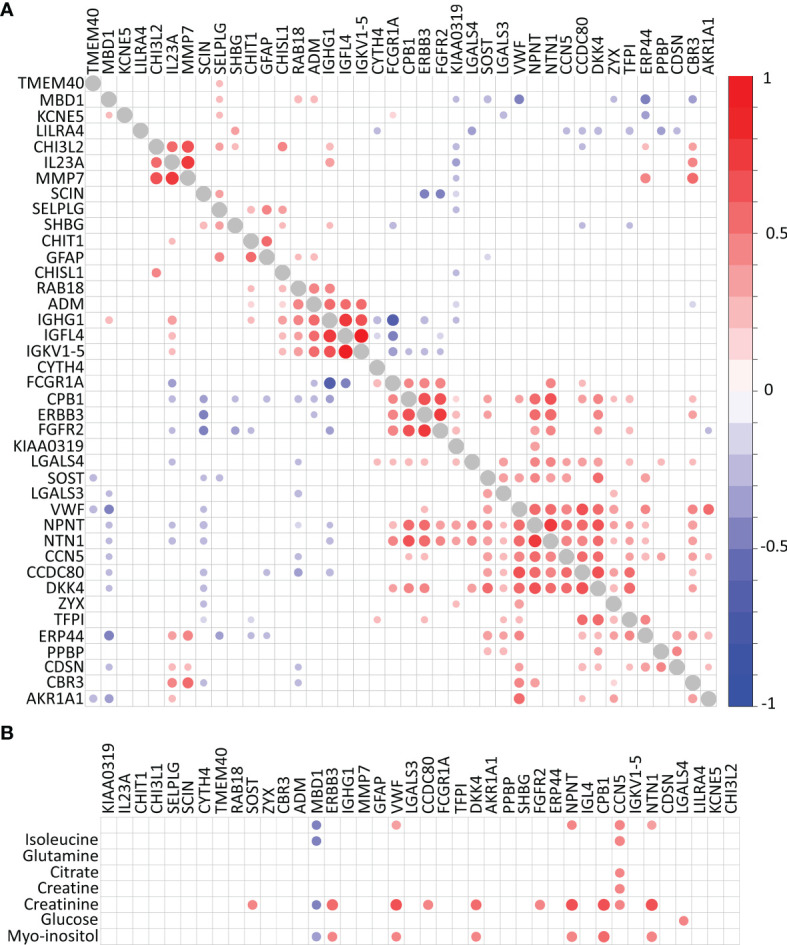
Correlation plots. **(A)** Protein-protein correlations and **(B)** metabolite-protein correlations. Correlations with self (diagonal) represented in grey. Significant Pearson’s R correlations prior to multiple comparison correction are displayed below the diagonal while those which remain significant following correction for multiple comparisons are above the diagonal.

Hierarchical clustering reveals four highly correlated groups of proteins within the 40 biomarkers identified which separate the CDMS patients from non-MS independently of OCGB status (**Figure 3A**). In contrast, fewer significant correlations were observed between the metabolite and protein hits (**Figure 3B**). No correlations were observed between any of the metabolite hits and IgG associated proteins, consistent with our earlier observation that the identified discriminatory metabolites are independent of OCGB status.

**Figure 4:**
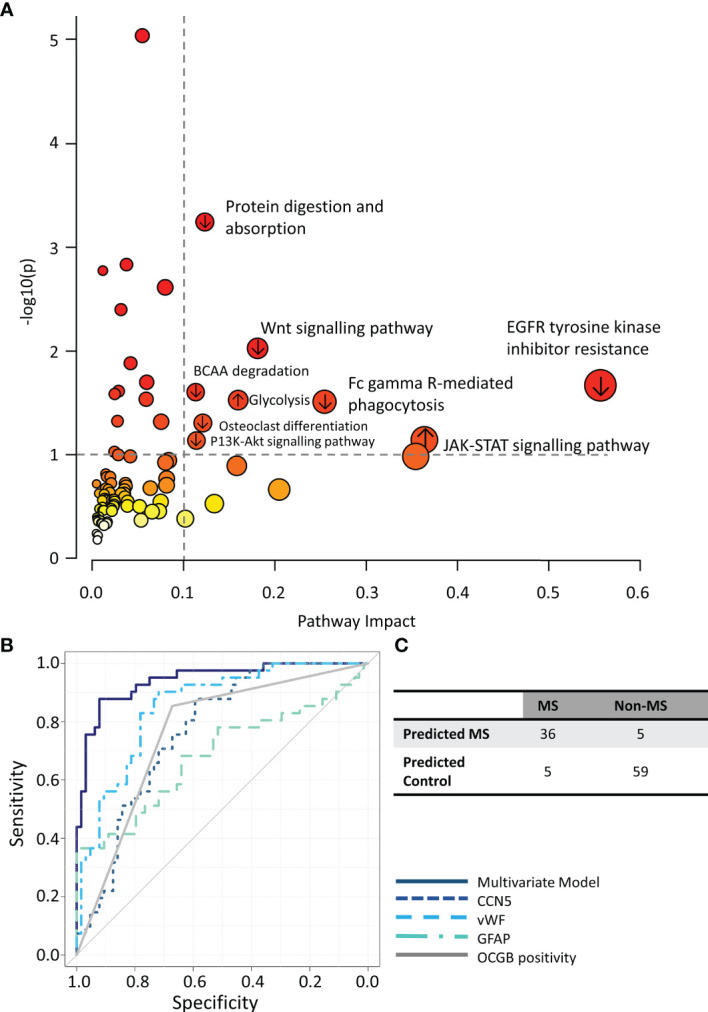
**(A)** Integrated metabolomics and proteomics pathway analysis. **(B)** ROC curves illustrating the performance of the multivariate model (solid navy) compared to each component of the model alone. **(C)** Confusion matrix of multiomics model predictions. ↑ pathways containing proteins/metabolites upregulated in CDMS, ↓ pathways containing proteins/metabolites downregulated in CDMS.

Integrative metabolomics and proteomics enrichment analysis revealed several potentially perturbed pathways in the CDMS group (**Figure 4A**). Of note, upregulation of the JAK-STAT and glycolysis pathways is consistent with an increased inflammatory response and perturbed energy metabolism in the CDMS cohort.

### A Combination of CCN5, vWF, and GFAP CSF Levels Discriminates Between OCGB+ve MS and OCGB+ve Non-MS With 91% Accuracy

Finally, we investigated if a multivariate diagnostic test combining proteomics, metabolomics, and OCGB measures could improve the diagnosis of MS. A combination of CCN5, vWF, GFAP, and OCGB status provided the greatest overall accuracy when discriminating between CDMS and non-MS CSF samples (**Figures S5A**–**D**). Indeed, OPLS-DA analysis using only these 4 variables resulted in an accuracy, sensitivity, and specificity or 91%, 89%, and 92% respectively on independent data which is significantly higher than the accuracy, sensitivity, and specificity of OCGB alone (+16%, +4%, and +25% respectively). The multivariate model out-performed, not only OCGB status, but all identified metabolite and protein biomarkers when measured in isolation (**Figure 4B**). For the OCGB-ve patients, the multiomics model correctly identified one (15%) of OCGB-ve CDMS patient as MS while all 43 (100%) OCGB-ve non-MS patients remained correctly identified as non-MS. However, the greatest clinical utility of this approach is in the identification of non-MS patients who test positive for OCGB (improved PPV). Indeed, the model correctly identified 16 (76%) OCGB+ve non-MS patients (**Figure 4C**) and 35 (100%) of OCGB+ve CDMS patients. This illustrates that the addition of the identified proteins to complement the use of OCGB status, within the context of the McDonald criteria, could greatly improve specificity and PPV without sacrificing sensitivity or NPV in the instance when DIT cannot be demonstrated radiologically or clinically, although this remains to be confirmed in a cohort of early MS patients. Interestingly, vWF may be substituted for either myo-inositol or TMEM40 in the multi-omics model with no significant drop in accuracy (**Table S3**)

## Discussion

Here, we have identified a small number of independent biomarkers, using feature selection coupled with a pattern recognition multivariate analysis framework and multiomics data, that may be used to support a diagnosis of MS with an accuracy of 90% (91% in the OCGB+ve patients and 90% in the OCGB-ve patients). The combination of CCN5, vWF, GFAP, and OCGB provides a significant increase in PPV; the model is able to accurately discriminate between OCGB+ve CDMS and OCGB+ve non-MS neurological conditions. While this combination yielded the highest accuracy, it is of note that other combinations could produce similar accuracies – for example, the substitution of myo-inositol for vWF. In each case, the levels of the newly identified biomarkers - CCN5, GFAP, and vWF - were independent of OCGB levels and outperformed OCGB alone.

CCN5 (previously known as WISP-2), which was decreased in the CSF of CDMS patients, is a member of the connective tissue growth factor/cysteine-rich 61/nephroblastoma overexpressed (CCN) family which play important roles in cell growth, adhesion, and migration. However, the function of CCN5 is not well understood. In an EAE model of MS, CCN5 mRNA was found to be significantly upregulated in spinal cord tissue, but as the tissue was collected in end-stage disease, its relevance to early-stage disease in MS is questionable (23). Interestingly, a significant positive correlation has been reported between levels of the related protein CCN3 in matched plasma and CSF of MS patients, which was absent in a comparator group of idiopathic intracranial hypertension patients (24). CCN3 plays various roles in the immune system and CCN5 and CCN3 have been reported to have antagonistic effects (25). CCN3 is a regulator of cytokine expression in both the periphery and CNS (26), where it can promote astrocyte activation (27), but it is not clear what effect CCN5 has on these processes.

The connection between the biology of multiple sclerosis and vWF, which was decreased in the CSF of CDMS patients compared to non-MS, is less opaque; vWF and/or Weibel-Palade bodies negatively regulate BBB permeability changes in MS-like lesions (28). It is not clear why the levels should be reduced in the CSF of MS patients, but one might speculate that increased release from endothelial cells into the blood might lead to a reduction in the CSF.

For GFAP, which was increased in the CDMS group, the connection with MS pathogenesis is now well established. Here, GFAP provided 100% specificity (+33% relative to OCGB), which suggests that measurement of this protein biomarker could be particularly useful in diagnosing MS in OCGB positive patients. GFAP is highly specific for astrocytic damage, and, as astrogliosis is a central component in MS pathogenesis, it is perhaps unsurprising that this protein should be highlighted in this analysis. However, it is important to note that other conditions have also been shown to be associated increase GFAP levels in the CSF. During acute NMO exacerbations, for example, CSF GFAP levels are significantly elevated (29) and after trauma (30). GFAP levels also increase with age, but, following an adjustment for age, MS patients have been shown to have higher GFAP levels compared with controls, and the adjusted levels correlate with neurological disability and disease progression (31). In studies on early MS, it has been reported that there are no significant differences in CSF GFAP levels between CIS and RRMS, but that GFAP does seem to be a good biomarker for highly active CNS inflammation in patients with CIS and RRMS (32). This would appear to highlight the need for a small panel of biomarkers rather than relying on one or another to aid in a diagnosis.

Myo-inositol is a component of all cell membranes and oligodendrocyte myelin and is involved in intracellular signalling in many CNS cell populations. It has been found to be increased in CSF in RRMS/CIS patients compared to healthy controls (33, 34), and in the brain of animals with EAE (35). The reduction in the CSF observed here could, therefore, reflect the sum difference between the anabolic and catabolic processes in MS versus other neurological disease where a relative loss in MS is present. Interestingly, myo-inositol levels can be used to accurately discriminate between RRMS and antibody mediated-NMOSD (36), where the further reduction in myo-inositol in antibody-mediated NMOSD may reflect demyelination and increased loss of astrocyte membrane. Hierarchical clustering revealed four highly correlated groups of proteins, but these did not fit with any known disease associated clusters. Pathway analysis revealed increased JAK-STAT signalling and glycolysis pathways in the MS cohort. The JAK-STAT pathway is activated by many cytokines, and its activation is key in almost all immune responses. The increase in glycolysis would be consistent with increased energy metabolism associated with inflammation.

For patients who are incorrectly diagnosed with MS, 50% have been found to carry the misdiagnosis for at least 3 years, and more than 5% were found to be misdiagnosed for over 20 years (37). This can lead to the administration of inappropriate and potentially harmful disease modifying therapies. Our results have shown how the inclusion of a small number of additional laboratory tests complement the high sensitivity of OCGB by increasing specificity and PPV and, thus, could significantly improve confidence in an MS diagnosis when DIT cannot be confirmed either radiologically or clinically. NMR metabolomics is a cheap and rapid analysis method, requiring minimal sample preparation and with the advantage that a host of additional small molecules can be quantified in the same analysis. Indeed, we have shown that NMR metabolomics is able to identify relapse (38), predict conversion (16), and diagnose progression (39) in MS and so, in future, it may be possible to apply multiple tests to a single sample. Future work will develop a chip-based assay to measure only the top biomarkers identified here in the hopes of improving translatability of this method. However, as CSF is routinely collected as part of the 2017 McDonald criteria, the addition of a small number of biomarker measurements to complement and improve the specificity of the already measured OCGB would be of benefit in a clinical setting. One limitation of this study might be considered to be that we used a cohort from one site which was limited in size (n=105). It is clear that a further prospective study in a larger independent cohort, collected across multiple centres, of early MS/clinically isolated syndrome patients, focused on the principal metabolite and protein biomarkers identified here, is now warranted. Should the results be conserved in a larger and broad population, the use of these markers in addition to OCGB could provide a valuable new diagnostic test for the presence of MS.

## Data Availability Statement

The datasets presented in this article are not readily available because they contain identifiable information from human subjects that cannot be shared in open access repositories for legal reasons. Anonymized data will be shared upon request from any qualified investigator. Requests should be directed to the corresponding authors.

## Ethics Statement

The studies involving human participants were reviewed and approved by University Hospital Basel local ethics committee. The patients/participants provided their written informed consent to participate in this study.

## Author Contributions

FP was involved in the design/conception of the study, performed the NMR data acquisition, developed in house R scripts, performed and interpreted the analysis of the results, and drafted the manuscript. TY was involved in the design/conception of the study, preparation of samples for NMR data acquisition, interpretation of results, and contributed to writing the manuscript. YZ contributed to sample preparation and NMR data acquisition. MS contributed to sample preparation and NMR data acquisition. SA contributed to data analysis design and preparation of the manuscript. JP was involved in the design/conception of the study and interpretation of results. TC was involved in acquisition and interpretation of NMR data. RH acquired the proteomics data. JO was involved in patient recruitment, clinical data acquisition, interpretation of results, and manuscript preparation. JK was involved in the design/conception of the study, was involved in clinical data acquisition, interpretation of results, and manuscript preparation. DL was involved in the design/conception of the study, was involved in clinical data acquisition, interpretation of results, and manuscript preparation. DA was involved in the design/conception of the study, was involved in clinical data acquisition, interpretation of results, and was a major contributor to writing the manuscript. All authors contributed to the article and approved the submitted version.

## Funding

Funding for the project and the salary of MS was provided as part of a GMSI grant award to the University of Oxford from Merck. The funder was not involved in the study design, collection, analysis, interpretation of data, the writing of this article or the decision to submit it for publication. FP received funding from the Multiple Sclerosis Society UK (grant 59) and the Medical Research Council (MC_PC_15029). TY is supported by the Ministry of Health, Singapore through the National Medical Research Council Research Training Fellowship (NMRC/Fellowship/0038/2016). FP received funding from the Multiple Sclerosis Society UK (grant 59), the Medical Research Council (MC_PC_15029) and a Dorothy Hodgkin Career Development Fellowship in Association with Somerville College.

## Conflict of Interest

Author RH was employed by company Novartis Pharma AG. DA received speaker fees, research support, travel support, and/or served on advisory boards Multiple Sclerosis Society UK, Merck, Novartis, and Roche. FP has received travel awards from ECTRIMS, Merck, and the Multiple Sclerosis Society UK. JK received speaker fees, research support, travel support, and/or served on advisory boards by ECTRIMS, Swiss MS Society, Swiss National Research Foundation, (320030_160221), University of Basel, Bayer, Biogen, Celgene, Merck, Novartis, Roche, Sanofi. TY has received travel grants from UCB, Merck and PACTRIMS, and travel awards from ECTRIMS, ACTRIMS and Orebro University. JP is partly funded by highly specialized services to run a national congenital myasthenia service and a neuromyelitis service. She has received support for scientific meetings and honorariums for advisory work from Merck Serono, Biogen Idec, Novartis, Teva, Chugai Pharma and Bayer Schering, Alexion, Roche, Genzyme, MedImmune, EuroImmun, MedDay, Abide ARGENX, UCB and Viela Bio and grants from Merck Serono, Novartis, Biogen Idec, Teva, Abide, MedImmune, Bayer Schering, Genzyme, Chugai and Alexion. She has received grants from the MS society, Guthrie Jackson Foundation, NIHR, Oxford Health Services Research Committee, EDEN, MRC, GMSI, John Fell and Myaware for research studies.

The remaining authors declare that the research was conducted in the absence of any commercial or financial relationships that could be construed as a potential conflict of interest.

## Publisher’s Note

All claims expressed in this article are solely those of the authors and do not necessarily represent those of their affiliated organizations, or those of the publisher, the editors and the reviewers. Any product that may be evaluated in this article, or claim that may be made by its manufacturer, is not guaranteed or endorsed by the publisher.
