# Corrigendum: Determination of CSF GFAP, CCN5, and vWF levels enhances the diagnostic accuracy of clinically defined MS from non-MS patients with CSF oligoclonal bands

**DOI:** 10.3389/fimmu.2022.1095038

**Published:** 2022-12-13

**Authors:** Fay Probert, Tianrong Yeo, Yifan Zhou, Megan Sealey, Siddharth Arora, Jacqueline Palace, Timothy D. W. Claridge, Rainer Hillenbrand, Johanna Oechtering, Jens Kuhle, David Leppert, Daniel C. Anthony

**Affiliations:** ^1^ Department of Chemistry, University of Oxford, Oxford, United Kingdom; ^2^ Department of Pharmacology, University of Oxford, Oxford, United Kingdom; ^3^ Department of Neurology, National Neuroscience Institute, Singapore, Singapore; ^4^ Duke-National University of Singapore (NUS) Medical School, Singapore, Singapore; ^5^ Translational Stem Cell Biology Branch, National Institutes of Health, Bethesda, MD, United States; ^6^ Wellcome Medical Research Council (MRC) Trust Stem Cell Institute, University of Cambridge, Cambridge, United Kingdom; ^7^ Department of Mathematics, University of Oxford, Oxford, United Kingdom; ^8^ Nuffield Department of Clinical Neurosciences, John Radcliffe Hospital, University of Oxford, Oxford, United Kingdom; ^9^ Biomarker Development, Novartis Pharma AG, Basel, Switzerland; ^10^ Neurologic Clinic and Policlinic, Multiple Sclerosis (MS) Center and Research Center for Clinical Neuroimmunology and Neuroscience Basel (RC2NB), Departments of Clinical Research and Biomedicine, University Hospital Basel, University of Basel, Basel, Switzerland

**Keywords:** diagnosis, metabolomics (OMICS), multiple sclerosis (MS), proteomics, oligoclonal band, biomarker

In the published article, there was an error in the Data Availability statement which was: "The datasets presented in this study can be found in online repositories. The names of the repository/repositories and accession number(s) can be found in the article/Supplementary Material". The correct Data Availability statement appears below.

## Data availability statement

The datasets presented in this article are not readily available because they contain identifiable information from human subjects that cannot be shared in open access repositories for legal reasons. Anonymized data will be shared upon request from any qualified investigator. Requests should be directed to the corresponding authors.

The authors apologize for this error and state that this does not change the scientific conclusions of the article in any way. The original article has been updated.

