# Supplementary material for: Determination of CSF GFAP, CCN5, and vWF Levels Enhances the Diagnostic Accuracy of Clinically Defined MS From Non-MS Patients With CSF Oligoclonal Bands
Source: Front Immunol. 2022 Feb 4;12:811351. doi: 10.3389/fimmu.2021.811351 (PMC8855362; doi:10.3389/fimmu.2021.811351)
Supplement: Supplementary file 1 [file DataSheet_1.pdf]

Determination of CSF GFAP, CCN5, and vWF levels enhances the diagnostic accuracy of clinically defined MS from non-MS patients with CSF oligoclonal bands

Fay Probert[1†*], Tianrong Yeo[2,3,4†], Yifan Zhou[2,5,6], Megan Sealey[2], Siddharth Arora[7], Jacqueline Palace[8], Timothy DW Claridge[1], Rainer Hillenbrand[9], Johanna Oechtering[10], Jens Kuhle[10], David Leppert[10], Daniel C Anthony[2*].

† these authors contributed equally

[1]Department of Chemistry, University of Oxford, UK

[2]Department of Pharmacology, University of Oxford, UK.

[3]Department of Neurology, National Neuroscience Institute, Singapore.

[4]Duke-NUS Medical School, Singapore

[5]Translational Stem Cell Biology Branch, National Institutes of Health, USA.

[6]Wellcome MRC Trust Stem Cell Institute, University of Cambridge, UK.

[7]Department of Mathematics, University of Oxford, UK.

[8]Nuffield Department of Clinical Neurosciences, John Radcliffe Hospital, University of Oxford, UK.

[9]Novartis Pharma AG, Basel, Switzerland.

[10]Neurologic Clinic and Policlinic, MS Center and Research Center for Clinical Neuroimmunology and Neuroscience Basel (RC2NB), Departments of Clinical Research and Biomedicine, University Hospital Basel, University of Basel, Switzerland.

*** Correspondence:** Daniel Anthony (daniel.anthony@pharm.ox.ac.uk) and Fay Probert (fay.probert@pharm.ox.ac.uk)

Keywords: multiple sclerosis, diagnosis, metabolomics, proteomics, oligoclonal bands, biomarker

A **Discrimination between MS and Non-MS is independent of non-MS diagnosis**

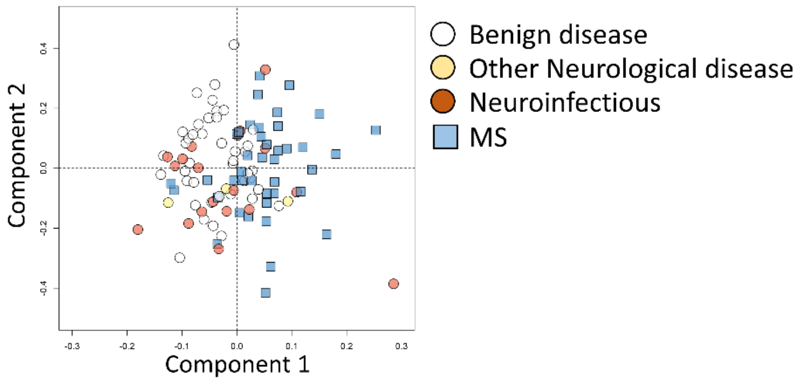

B **Discrimination between MS and Non-MS is independent of OCGB status**

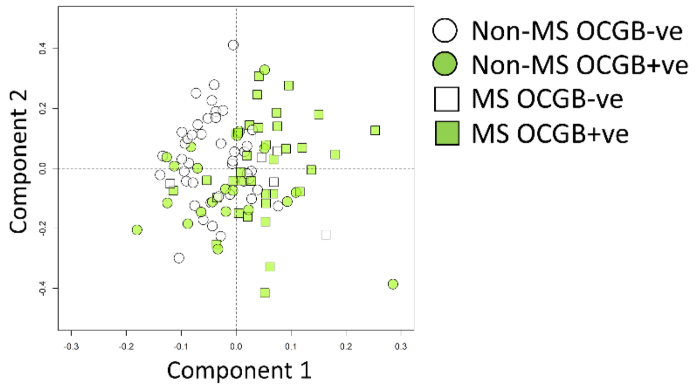

C **Discrimination between MS and Non-MS is independent of Age**

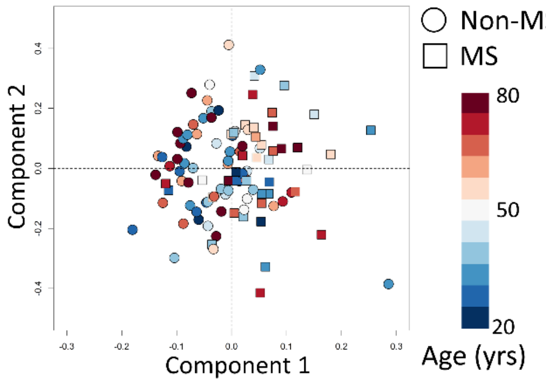

D **Discrimination between CDMS and Non-MS is independent of gender**

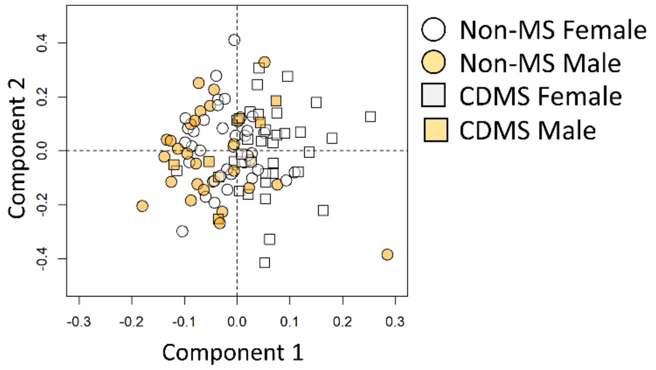

Supplementary Fig. 1 OPLS-DA scores plots discriminating between CDMS (squares) and non-MS (circles) using metabolomics data alone. Points are coloured by a) non-MS diagnostic group, b) OCGB status, c) age, and d) age. All potential confounders are equally distributed to the left and right of the scores plot illustrating that the model is able to identify MS versus non-MS patients independently of the non-MS diagnosis, OCGB status, age, or gender. Of note, there is no separation of gender within the non-MS control (circles) cluster despite a more even gender distribution in this class.

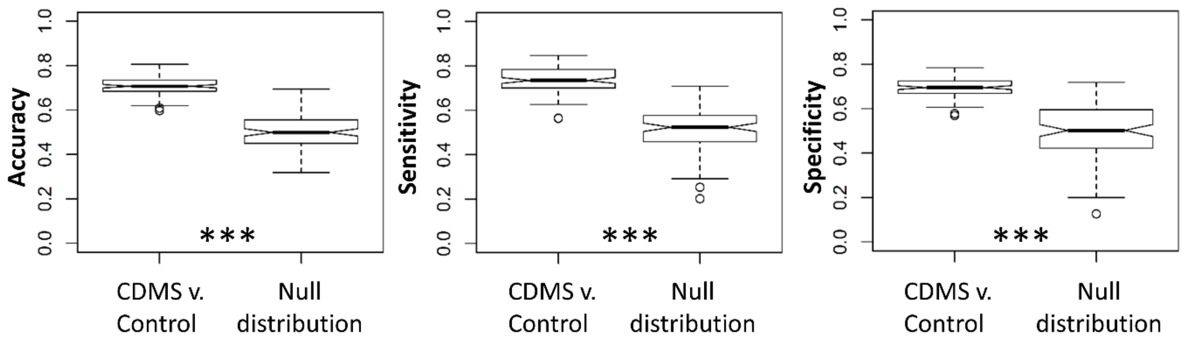

Supplementary Fig. 2 Validating metabolomics OPLS-DA models on independent test data. 10-fold cross-validation with repetition reveals significantly increased accuracy, sensitivity, and specificity on independent test data (excluded when training the model) relative to the null distribution produced by permutation testing (the performance expected by random chance alone). Two-sample Kolmogorov-Smirnov p-values < 0.001 are represented by ***

A Discrimination between MS and Non-MS is independent of non-MS diagnosis

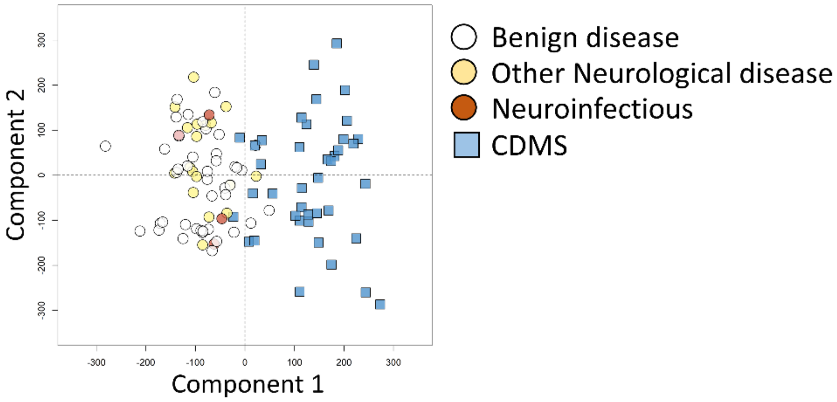

B Discrimination between MS and Non-MS is independent of OCGB status

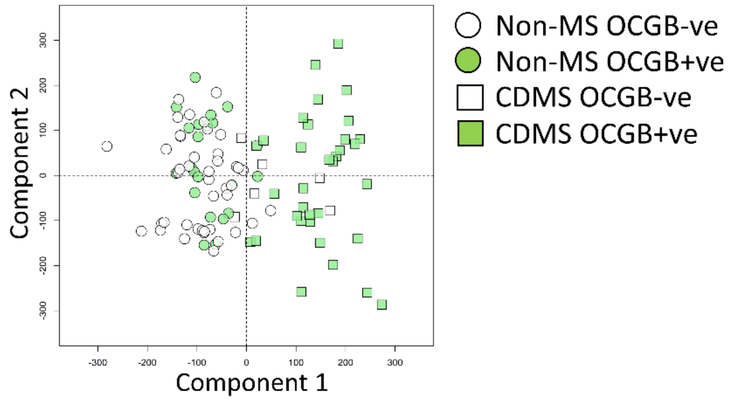

C Discrimination between MS and Non-MS is independent of Age

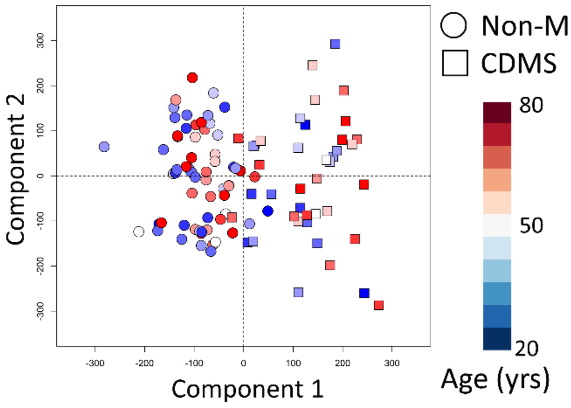

D Discrimination between MS and Non-MS is independent of gender

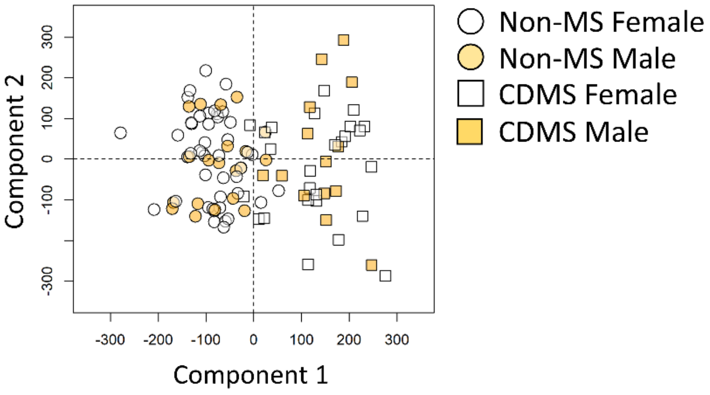

Supplementary Fig. 3 OPLS-DA scores plots discriminating between MS (squares) and non-MS (circles) using proteomics data alone. Points are coloured by a) non-MS diagnostic group, b) OCGB status, c) age, and d) gender. All potential confounders are equally distributed to the left and right of the scores plot illustrating that the model is able to identify MS versus non-MS patients independently of the non-MS diagnosis, OCGB status, age, or gender. Of note, there is no separation of gender within the non-MS control (circles) cluster despite a more even gender distribution in this class.

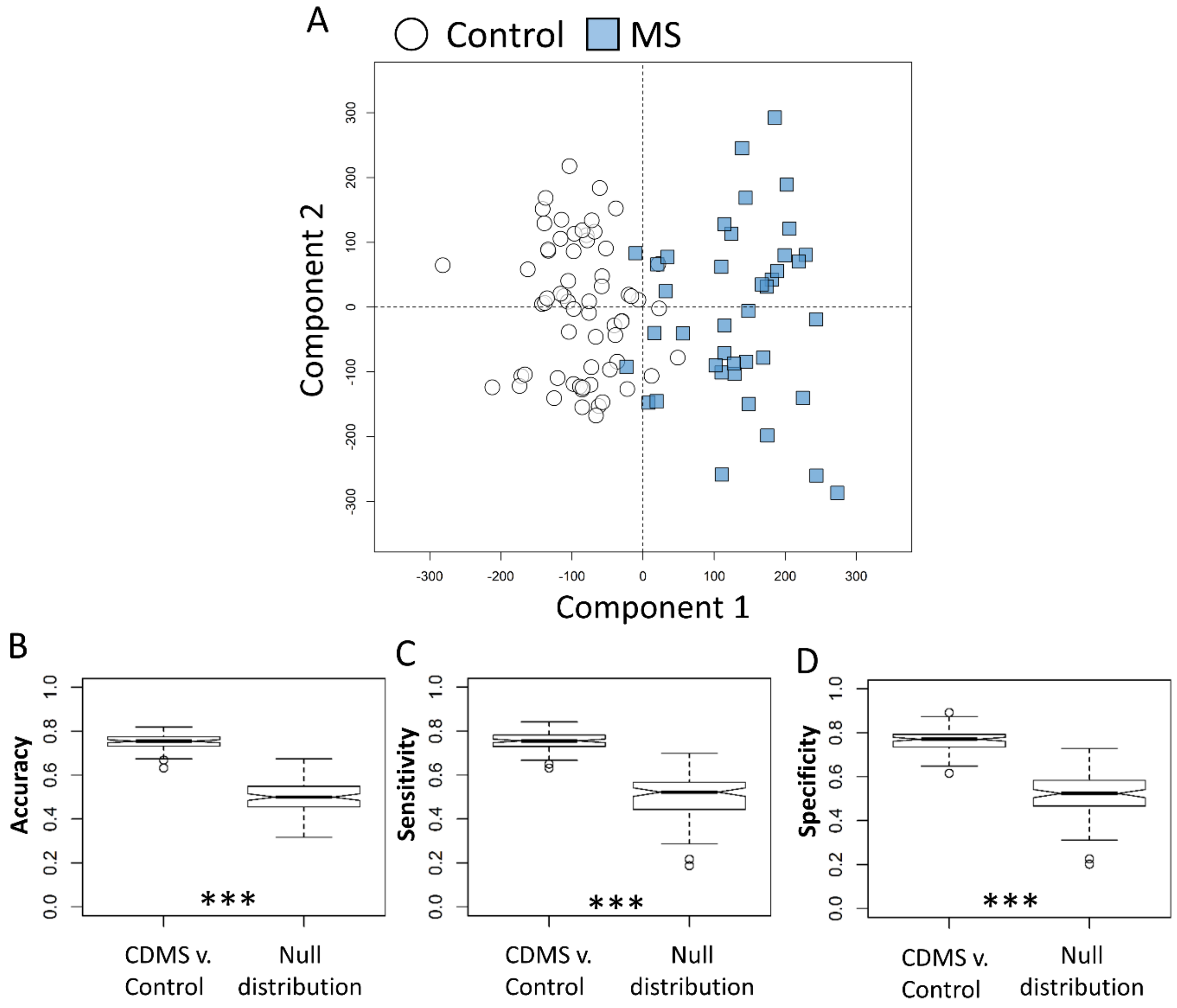

Supplementary Fig. 4 Validating proteomics OPLS-DA models on independent test data. a) Representative OPLS-DA scores plot illustrating discrimination between MS (blue square, n=41) and Control (white circle, n=64) CSF proteomics profiles. 10-fold cross-validation with repetition reveals significantly increased b) accuracy, c) sensitivity, and d) specificity on independent test data (excluded when training the model) relative to the null distribution produced by permutation testing (the performance expected by random chance alone). Two-sample Kolmogorov-Smirnov p-values < 0.001 are represented by ***

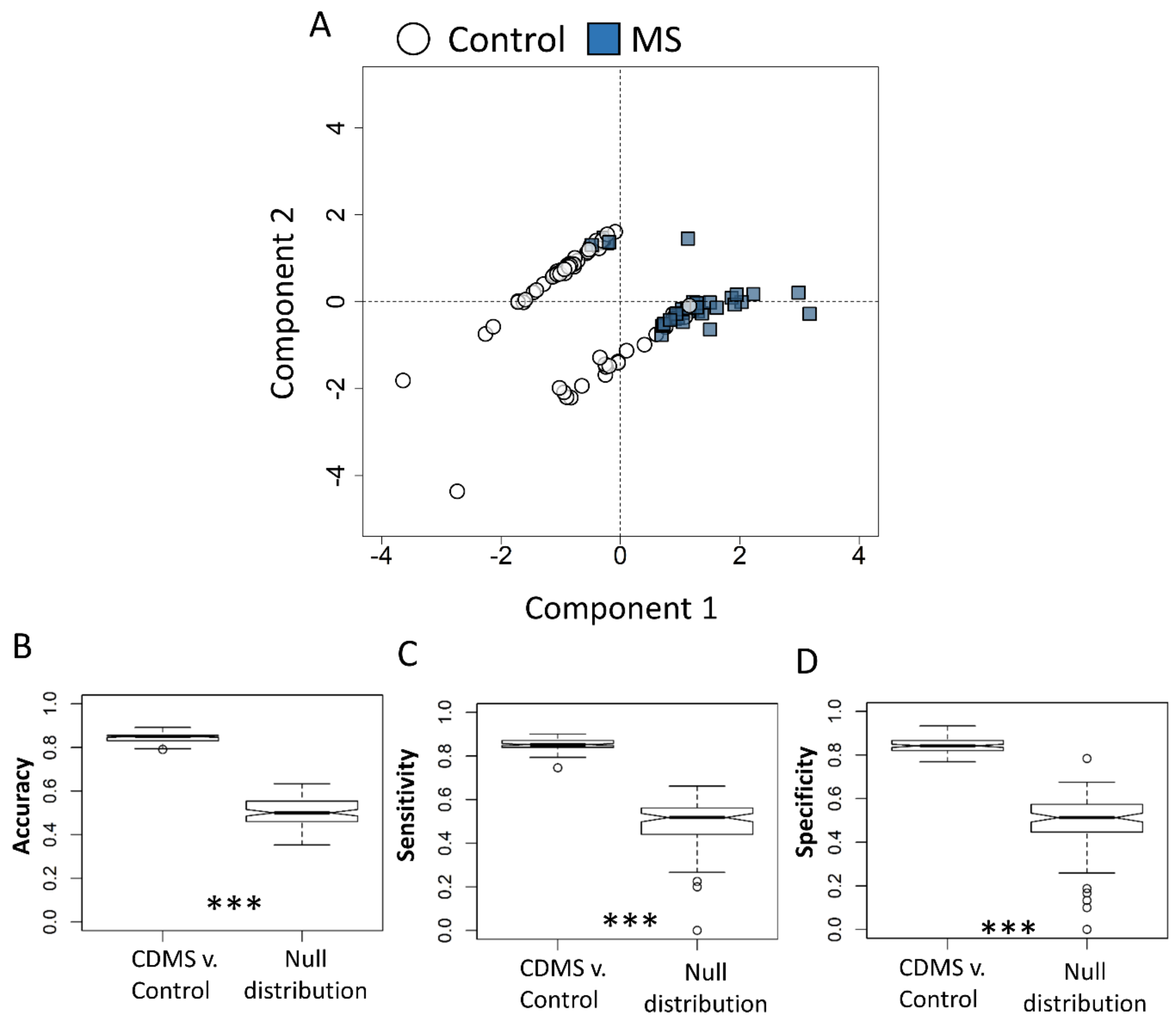

Supplementary Fig. 5 Validating multi-omics OPLS-DA models on independent test data. a) Representative OPLS-DA scores plot illustrating discrimination between MS (grey square, n=41) and Control (white circle, n=64) CSF proteomics profiles. 10-fold cross-validation with repetition reveals significantly increased b) accuracy, c) sensitivity, and d) specificity on independent test data (excluded when training the model) relative to the null distribution produced by permutation testing (the performance expected by random chance alone). Two-sample Kolmogorov-Smirnov p-values < 0.001 are represented by ***

Supplementary Table 1. List of significant proteins identified by OPLS-DA which drive the discrimination between MS and Controls ranked from highest to lowest AUC. 2-way ANOVA p-values less than 0.001, 0.01, and 0.05 following Bonferroni correction for multiple comparisons are represented by ***, **, and * respectively. ns; not significant, ↓; decrease in MS relative to Control. Diagnostic accuracy of OCGB status is included for comparison. AUC; receiver operator curve area under the curve, PPV; positive predictive value, NPV; negative predictive value, OCGB; CSF oligoclonal bands, Acc; accuracy, Sens; sensitivity, Spec; specificity, PPV; positive predictive value, NPV; negative predictive value, TP; true positive, FN, false negative; FP; false positive, TN; true negative.

Uniprot #	Gene	Protein	MS v Control (fold change)	OCGB+ve v. OCGB-ve [p-value]	Interaction [p-value]	AUC	Acc (%)	Sens (%)	Spec (%)	PPV (%)	NPV (%)	TP	FN	FP	TN
O76076	CCN5	CCN family member 5 (WISP-2)	↓*** (0.61)	ns [3.18]	ns [4.72]	0.85	79	90	72	67	92	37	4	18	46
Q76M96	CCDC80	Coiled-coil domain-containing protein 80	↓*** (0.79)	ns [30.58]	ns [3.21]	0.83	76	71	80	69	81	29	12	13	51
Q6B9Z1	IGFL4	Insulin growth factor-like family member 4	↑ns (1.57)	ns [0.1]	ns [27.79]	0.81	80	73	84	75	83	30	11	10	54
O95631	NTN1	Netrin-1	↓*** (0.86)	ns [32.8]	ns [23.25]	0.81	73	85	66	61	88	35	6	22	42
P01602	IGKV1-5	Immunoglobulin kappa variable 1-5	↑ns (1.24)	ns [0.91]	ns [39.39]	0.8	81	68	89	80	81	28	13	7	57

P01857	IGHG1	Immunoglobulin heavy constant gamma 1	↑*** (1.79)	*** [<0.001]	ns [14.77]	0.77	77	76	78	69	83	31	10	14	50
Q9NPF7	IL23A	Interleukin-23 subunit alpha	↑ns (1.51)	** [0.006]	ns [14.39]	0.77	78	80	77	69	86	33	8	15	49
Q6UXI9	NPNT	Nephronectin	↓*** (0.82)	ns [30.61]	ns [34.37]	0.77	73	76	72	63	82	31	10	18	46
P04275	VWF	von Willebrand factor (vWF)	↓*** (0.63)	ns [0.45]	ns [6.22]	0.77	70	88	59	58	88	36	5	26	38
Q9UBT3	DKK4	Dickkopf-related protein 4	↓*** (0.59)	ns [37.37]	ns [13.99]	0.76	70	90	56	57	90	37	4	28	36
Q9BS26	ERP44	Endoplasmic reticulum resident protein 44	↓** (0.83)	ns [1.01]	ns [0.17]	0.75	73	83	67	62	86	34	7	21	43
P21860	ERBB3	Receptor tyrosine-protein kinase erbB-3	↓** (0.81)	ns [11.22]	ns [22.35]	0.75	71	78	67	60	83	32	9	21	43
Q9BQB4	SOST	Sclerostin	↓** (0.78)	ns [15.21]	ns [14.8]	0.75	68	80	59	56	83	33	8	26	38
O75828	CBR3	NADPH-dependent carbonyl reductase 3	↓* (0.71)	ns [3.75]	ns [2.89]	0.74	72	66	77	64	78	27	14	15	49
NA	NA	OCGB	NA	NA	NA	0.74	74	85	67	63	88	35	6	21	43
P15086	CPB1	Carboxypeptidase B	↓*** (0.70)	ns [2.18]	ns [26.24]	0.74	69	85	58	56	86	35	6	27	37

P10646	TFPI	Tissue factor pathway inhibitor (TFPI)	↓** (0.80)	ns [7.23]	ns [0.18]	0.73	74	54	88	73	75	22	19	8	56
P12314	FCGR1A	High affinity immunoglobulin gamma Fc receptor I	↓*** (0.77)	** [0.008]	ns [25.85]	0.73	72	56	83	68	75	23	18	11	53
P17931	LGALS3	Galectin-3	↓** (0.90)	ns [13.21]	ns [32.75]	0.73	73	68	77	65	79	28	13	15	49
P59901	LILRA4	Leukocyte immunoglobulin-like receptor subfamily A member 4	↑** (1.17)	ns [3.21]	ns [39.33]	0.72	72	63	78	65	77	26	15	14	50
P35318	ADM	Pro-adrenomedullin	↑* (1.07)	ns [11.39]	ns [24.87]	0.72	70	68	72	61	78	28	13	18	46
Q15782	CHI3L2	Chitinase-3-like protein 2	↑* (1.37)	ns [9.82]	ns [12.55]	0.72	68	71	66	57	78	29	12	22	42
Q9NP72	RAB18	Ras-related protein Rab-18	↑** (1.13)	ns [1.92]	ns [2.75]	0.71	73	63	80	67	77	26	15	13	51
Q5VV43	KIAA0319	Dyslexia-associated protein KIAA0319	↑* (1.06)	ns [12.69]	ns [30.38]	0.71	69	63	72	59	75	26	15	18	46
P04278	SHBG	Sex hormone-binding globulin (SHBG)	↑* (1.44)	ns [16]	ns [11.14]	0.71	68	71	66	57	78	29	12	22	42
P56470	LGALS4	Galectin-4	↓** (0.73)	ns [7.77]	ns [33.55]	0.71	65	73	59	54	78	30	11	26	38

P21802	FGFR2	Fibroblast growth factor receptor 2 (FGFR-2)	↓** (0.85)	ns [0.34]	ns [0.98]	0.71	67	80	58	55	82	33	8	27	37
Q9Y6U3	SCIN	Adseverin	↑** (1.13)	ns [19.66]	ns [33.64]	0.7	65	76	58	53	79	31	10	27	37
Q14242	SELPLG	P-selectin glycoprotein ligand 1	↑* (1.19)	ns [9.15]	ns [10.68]	0.69	68	68	67	57	77	28	13	21	43
Q15942	ZYX	Zyxin	↓* (0.91)	ns [37.02]	ns [23.45]	0.69	65	76	58	53	79	31	10	27	37
P14136	GFAP	Glial fibrillary acidic protein (GFAP)	↑** (2.34)	ns [3.39]	ns [27.65]	0.68	75	37	100	100	71	15	26	0	64
Q13231	CHIT1	Chitotriosidase-1 (Chitinase-1)	↑** (2.17)	ns [3.39]	ns [27.65]	0.68	75	68	80	68	80	28	13	13	51
Q15517	CDSN	Corneodesmosin (S protein)	↓ns (0.89)	ns [37.24]	ns [35.58]	0.68	61	88	44	50	85	36	5	36	28
P36222	CHI3L1	Chitinase-3-like protein 1	↑ns (1.29)	ns [10.01]	ns [0.92]	0.67	69	54	78	61	72	22	19	14	50
P14550	AKR1A1	Aldo-keto reductase family 1 member A1	↓ns (0.83)	** [0.002]	ns [38.89]	0.67	66	66	66	55	75	27	14	22	42
Q9UIS9	MBD1	Methyl-CpG-binding domain protein 1	↑* (1.17)	ns [13.6]	ns [7.01]	0.65	72	49	88	71	73	20	21	8	56
P09237	MMP7	Matrilysin	↑ns (1.03)	ns [3.54]	ns [16.38]	0.65	63	71	58	52	76	29	12	27	37
Q9UIA0	CYTH4	Cytohesin-4	↓ns (0.86)	ns [1.99]	ns [29.56]	0.64	67	54	75	58	72	22	19	16	48
P02775	PPBP	Platelet basic protein (PBP)	↓ns (0.75)	ns [38.88]	ns [27.04]	0.63	54	93	30	46	86	38	3	45	19

Q8WWA1	TMEM40	Transmembrane protein 40	↑** (1.11)	ns [1.53]	ns [26.37]	0.61	71	37	94	79	70	15	26	4	60
Q9UJ90	KCNE5	Potassium voltage-gated channel subfamily E regulatory beta subunit 5	↑ns (1.09)	ns [1.58]	ns [1.55]	0.54	54	80	38	45	75	33	8	40	24

Supplementary Table 2. List of significant proteins identified by OPLS-DA which drive the discrimination between MS and Controls with greater specificity than OCGB. 2-way ANOVA p-values less than 0.001, 0.01, and 0.05 following Bonferroni correction for multiple comparisons are represented by ***, **, and * respectively. ns; not significant, ↓; decrease in MS relative to Control. Diagnostic accuracy of OCGB status is included for comparison. AUC; receiver operator curve area under the curve, PPV; positive predictive value, NPV; negative predictive value, OCGB; CSF oligoclonal bands, Acc; accuracy, Sens; sensitivity, Spec; specificity, PPV; positive predictive value, NPV; negative predictive value, TP; true positive, FN, false negative; FP; false positive, TN; true negative.

**Uniprot #**	**Gene**	**Protein**	**MS v Control (fold change)**	**OCGB+ve v. OCGB-ve [p-value]**	**Interaction [p-value]**	**AUC**	**Acc (%)**	**Sens (%)**	**Spec (%)**	**PPV (%)**	**NPV (%)**	**TP**	**FN**	**FP**	**TN**
P14136	GFAP	Glial fibrillary acidic protein (GFAP)	↑** (2.34)	ns [3.39]	ns [27.65]	0.68	75	37	100	100	71	15	26	0	64
Q8WWA1	TMEM40	Transmembrane protein 40	↑** (1.11)	ns [1.53]	ns [26.37]	0.61	71	37	94	79	70	15	26	4	60
P01602	IGKV1-5	Immunoglobulin kappa variable 1-5	↑ns (1.24)	ns [0.91]	ns [39.39]	0.8	81	68	89	80	81	28	13	7	57
P10646	TFPI	Tissue factor pathway inhibitor (TFPI)	↓** (0.80)	ns [7.23]	ns [0.18]	0.73	74	54	88	73	75	22	19	8	56
Q9UIS9	MBD1	Methyl-CpG-binding domain protein 1	↑* (1.17)	ns [13.6]	ns [7.01]	0.65	72	49	88	71	73	20	21	8	56
Q6B9Z1	IGFL4	Insulin growth factor-like family member 4	↑ns (1.57)	ns [0.1]	ns [27.79]	0.81	80	73	84	75	83	30	11	10	54
P12314	FCGR1A	High affinity immunoglobulin gamma Fc receptor I	↓*** (0.77)	** [0.008]	ns [25.85]	0.73	72	56	83	68	75	23	18	11	53

Q76M96	CCDC80	Coiled-coil domain-containing protein 80	↓*** (0.79)	ns [30.58]	ns [3.21]	0.83	76	71	80	69	81	29	12	13	51
Q9NP72	RAB18	Ras-related protein Rab-18	↑** (1.13)	ns [1.92]	ns [2.75]	0.71	73	63	80	67	77	26	15	13	51
Q13231	CHIT1	Chitotriosidase-1 (Chitinase-1)	↑** (2.17)	ns [3.39]	ns [27.65]	0.68	75	68	80	68	80	28	13	13	51
P01857	IGHG1	Immunoglobulin heavy constant gamma 1	↑*** (1.79)	*** [<0.001]	ns [14.77]	0.77	77	76	78	69	83	31	10	14	50
P59901	LILRA4	Leukocyte immunoglobulin-like receptor subfamily A member 4	↑** (1.17)	ns [3.21]	ns [39.33]	0.72	72	63	78	65	77	26	15	14	50
P36222	CHI3L1	Chitinase-3-like protein 1	↑ns (1.29)	ns [10.01]	ns [0.92]	0.67	69	54	78	61	72	22	19	14	50
Q9NPF7	IL23A	Interleukin-23 subunit alpha	↑ns (1.51)	** [0.006]	ns [14.39]	0.77	78	80	77	69	86	33	8	15	49
O75828	CBR3	NADPH-dependent carbonyl reductase 3	↓* (0.71)	ns [3.75]	ns [2.89]	0.74	72	66	77	64	78	27	14	15	49
P17931	LGALS3	Galectin-3	↓** (0.90)	ns [13.21]	ns [32.75]	0.73	73	68	77	65	79	28	13	15	49
Q9UIA0	CYTH4	Cytohesin-4	↓ns (0.86)	ns [1.99]	ns [29.56]	0.64	67	54	75	58	72	22	19	16	48
O76076	CCN5	CCN family member 5 (WISP-2)	↓*** (0.61)	ns [3.18]	ns [4.72]	0.85	79	90	72	67	92	37	4	18	46
Q6UXI9	NPNT	Nephronectin	↓*** (0.82)	ns [30.61]	ns [34.37]	0.77	73	76	72	63	82	31	10	18	46
P35318	ADM	Pro-adrenomedullin	↑* (1.07)	ns [11.39]	ns [24.87]	0.72	70	68	72	61	78	28	13	18	46

Q5VV43	KIAA0319	Dyslexia-associated protein KIAA0319	↑* (1.06)	ns [12.69]	ns [30.38]	0.71	69	63	72	59	75	26	15	18	46
Q9BS26	ERP44	Endoplasmic reticulum resident protein 44	↓** (0.83)	ns [1.01]	ns [0.17]	0.75	73	83	67	62	86	34	7	21	43
P21860	ERBB3	Receptor tyrosine-protein kinase erbB-3	↓** (0.81)	ns [11.22]	ns [22.35]	0.75	71	78	67	60	83	32	9	21	43
NA	NA	OCGB	NA	NA	NA	0.74	74	85	67	63	88	35	6	21	43
Q14242	SELPLG	P-selectin glycoprotein ligand 1	↑* (1.19)	ns [9.15]	ns [10.68]	0.69	68	68	67	57	77	28	13	21	43
O95631	NTN1	Netrin-1	↓*** (0.86)	ns [32.8]	ns [23.25]	0.81	73	85	66	61	88	35	6	22	42
Q15782	CHI3L2	Chitinase-3-like protein 2	↑* (1.37)	ns [9.82]	ns [12.55]	0.72	68	71	66	57	78	29	12	22	42
P04278	SHBG	Sex hormone-binding globulin (SHBG)	↑* (1.44)	ns [16]	ns [11.14]	0.71	68	71	66	57	78	29	12	22	42
P14550	AKR1A1	Aldo-keto reductase family 1 member A1	↓ns (0.83)	** [0.002]	ns [38.89]	0.67	66	66	66	55	75	27	14	22	42
P04275	VWF	von Willebrand factor (vWF)	↓*** (0.63)	ns [0.45]	ns [6.22]	0.77	70	88	59	58	88	36	5	26	38
Q9BQB4	SOST	Sclerostin	↓** (0.78)	ns [15.21]	ns [14.8]	0.75	68	80	59	56	83	33	8	26	38
P56470	LGALS4	Galectin-4	↓** (0.73)	ns [7.77]	ns [33.55]	0.71	65	73	59	54	78	30	11	26	38
P15086	CPB1	Carboxypeptidase B	↓*** (0.70)	ns [2.18]	ns [26.24]	0.74	69	85	58	56	86	35	6	27	37
P21802	FGFR2	Fibroblast growth factor receptor 2 (FGFR-2)	↓** (0.85)	ns [0.34]	ns [0.98]	0.71	67	80	58	55	82	33	8	27	37

Q9Y6U3	SCIN	Adseverin	↑** (1.13)	ns [19.66]	ns [33.64]	0.7	65	76	58	53	79	31	10	27	37
Q15942	ZYX	Zyxin	↓* (0.91)	ns [37.02]	ns [23.45]	0.69	65	76	58	53	79	31	10	27	37
P09237	MMP7	Matrilysin	↑ns (1.03)	ns [3.54]	ns [16.38]	0.65	63	71	58	52	76	29	12	27	37
Q9UBT3	DKK4	Dickkopf-related protein 4	↓*** (0.59)	ns [37.37]	ns [13.99]	0.76	70	90	56	57	90	37	4	28	36
Q15517	CDSN	Corneodesmosin (S protein)	↓ns (0.89)	ns [37.24]	ns [35.58]	0.68	61	88	44	50	85	36	5	36	28
Q9UJ90	KCNE5	Potassium voltage-gated channel subfamily E regulatory beta subunit 5	↑ns (1.09)	ns [1.58]	ns [1.55]	0.54	54	80	38	45	75	33	8	40	24
P02775	PPBP	Platelet basic protein (PBP)	↓ns (0.75)	ns [38.88]	ns [27.04]	0.63	54	93	30	46	86	38	3	45	19

Supplementary Table 3. Variables selected by the top 100 multi-omics models ranked from highest to lowest accuracy (of OPLS-DA models determined on independent test data via 10-fold cross validation. Var.; variable, AUC; area under the curve.

Rank	Var.1	Var.2	Var.3	Var.4	Accuracy	AUC
1	GFAP	VWF	CCN5	OCGB	88.4	0.94
2	GFAP	CCN5	myoinositol	OCGB	87.5	0.93
3	GFAP	TMEM40	CCN5	OCGB	87.5	0.95
4	GFAP	VWF	glutamine	OCGB	87.4	0.93
5	DKK4	TMEM40	CCN5	OCGB	86.7	0.94
6	TMEM40	VWF	CCN5	OCGB	86.6	0.94
7	DKK4	GFAP	CCN5	OCGB	86.6	0.93
8	GFAP	CCN5	creatine	OCGB	86.5	0.94
9	VWF	CCN5	creatine	OCGB	86.5	0.93
10	VWF	CCN5	myoinositol	OCGB	86.5	0.92
11	VWF	CCN5	leucine	OCGB	86.5	0.91
12	GFAP	CCN5	creatinine	OCGB	86.5	0.93
13	GFAP	VWF	leucine	OCGB	86.4	0.93
14	GFAP	VWF	myoinositol	OCGB	86.4	0.92
15	TMEM40	CCN5	creatine	OCGB	85.7	0.94
16	DKK4	GFAP	creatine	OCGB	85.7	0.93
17	DKK4	GFAP	glutamine	OCGB	85.7	0.92
18	TMEM40	CCN5	creatinine	OCGB	85.6	0.94
19	IGKV1-5	VWF	CCN5	OCGB	85.6	0.92
20	DKK4	VWF	CCN5	OCGB	85.6	0.92
21	DKK4	TMEM40	VWF	OCGB	85.6	0.91
22	VWF	CCN5	glutamine	OCGB	85.5	0.93
23	TMEM40	VWF	creatine	OCGB	85.5	0.93
24	VWF	CCN5	creatinine	OCGB	85.5	0.92
25	GFAP	VWF	creatine	OCGB	85.5	0.94
26	DKK4	GFAP	VWF	OCGB	85.4	0.93
27	GFAP	TMEM40	VWF	OCGB	85.4	0.93
28	GFAP	VWF	creatinine	OCGB	85.4	0.92
29	TMEM40	CCN5	leucine	OCGB	84.8	0.93
30	DKK4	CCN5	creatine	OCGB	84.8	0.92
31	DKK4	CCN5	leucine	OCGB	84.8	0.91
32	DKK4	CCN5	myoinositol	OCGB	84.8	0.9
33	TMEM40	CCN5	myoinositol	OCGB	84.7	0.94
34	DKK4	GFAP	TMEM40	OCGB	84.7	0.94
35	IGFL4	VWF	CCN5	OCGB	84.7	0.92
36	CCN5	creatine	myoinositol	OCGB	84.7	0.92
37	DKK4	TMEM40	creatine	OCGB	84.7	0.92
38	CCN5	leucine	myoinositol	OCGB	84.7	0.91
39	GFAP	IGKV1-5	CCN5	OCGB	84.6	0.93
40	TMEM40	VWF	glutamine	OCGB	84.6	0.92
41	DKK4	TMEM40	creatinine	OCGB	84.6	0.92
42	IGKV1-5	TMEM40	VWF	OCGB	84.6	0.9

43	GFAP	CCN5	glutamine	OCGB	84.5	0.93
44	VWF	glutamine	creatine	OCGB	84.5	0.92
45	TMEM40	VWF	myoinositol	OCGB	84.5	0.91
46	VWF	creatine	creatinine	OCGB	84.5	0.91
47	VWF	glutamine	creatinine	OCGB	84.5	0.9
48	IGKV1-5	TMEM40	CCN5	OCGB	83.9	0.93
49	IGFL4	TMEM40	CCN5	OCGB	83.9	0.93
50	DKK4	IGKV1-5	CCN5	OCGB	83.9	0.91
51	DKK4	IGFL4	CCN5	OCGB	83.9	0.91
52	IGKV1-5	CCN5	myoinositol	OCGB	83.9	0.9
53	IGFL4	CCN5	myoinositol	OCGB	83.9	0.9
54	DKK4	CCN5	glutamine	OCGB	83.8	0.92
55	TMEM40	creatine	creatinine	OCGB	83.8	0.91
56	DKK4	CCN5	creatinine	OCGB	83.8	0.91
57	IGFL4	CCN5	leucine	OCGB	83.8	0.91
58	CCN5	leucine	creatine	OCGB	83.8	0.91
59	IGKV1-5	CCN5	leucine	OCGB	83.8	0.91
60	DKK4	creatine	creatinine	OCGB	83.8	0.9
61	GFAP	IGFL4	CCN5	OCGB	83.7	0.93
62	CCN5	creatine	creatinine	OCGB	83.7	0.92
63	DKK4	GFAP	myoinositol	OCGB	83.7	0.91
64	IGFL4	TMEM40	VWF	OCGB	83.7	0.9
65	DKK4	GFAP	TMEM40	leucine	83.7	0.89
66	IGKV1-5	VWF	creatine	OCGB	83.6	0.92
67	GFAP	creatine	creatinine	OCGB	83.6	0.91
68	TMEM40	VWF	creatinine	OCGB	83.6	0.91
69	DKK4	GFAP	creatinine	OCGB	83.6	0.91
70	GFAP	glutamine	creatinine	OCGB	83.6	0.9
71	IGFL4	VWF	myoinositol	OCGB	83.6	0.89
72	IGKV1-5	VWF	myoinositol	OCGB	83.6	0.89
73	IGFL4	VWF	creatinine	OCGB	83.6	0.89
74	DKK4	VWF	creatine	OCGB	83.5	0.92
75	GFAP	TMEM40	creatinine	OCGB	83.5	0.92
76	GFAP	IGKV1-5	VWF	OCGB	83.5	0.92
77	GFAP	IGFL4	VWF	OCGB	83.5	0.92
78	VWF	creatine	myoinositol	OCGB	83.5	0.91
79	GFAP	leucine	creatinine	OCGB	83.5	0.9
80	VWF	glutamine	myoinositol	OCGB	83.5	0.9
81	VWF	leucine	glutamine	OCGB	83.5	0.9
82	VWF	creatinine	myoinositol	OCGB	83.5	0.89
83	VWF	leucine	creatinine	OCGB	83.5	0.89
84	IGFL4	CCN5	creatinine	OCGB	82.9	0.92
85	IGKV1-5	CCN5	creatine	OCGB	82.9	0.91
86	IGFL4	CCN5	creatine	OCGB	82.9	0.91
87	IGKV1-5	CCN5	creatinine	OCGB	82.9	0.91
88	DKK4	IGKV1-5	TMEM40	OCGB	82.9	0.91

89	DKK4	GFAP	IGKV1-5	OCGB	82.9	0.91
90	IGKV1-5	IGFL4	CCN5	OCGB	82.9	0.91
91	DKK4	creatine	myoinositol	OCGB	82.9	0.9
92	GFAP	TMEM40	creatine	OCGB	82.8	0.93
93	CCN5	glutamine	creatinine	OCGB	82.8	0.92
94	GFAP	creatine	myoinositol	OCGB	82.8	0.91
95	TMEM40	creatine	myoinositol	OCGB	82.8	0.91
96	DKK4	TMEM40	myoinositol	OCGB	82.8	0.91
97	TMEM40	CCN5	glutamine	OCGB	82.7	0.94
98	IGFL4	VWF	creatine	OCGB	82.7	0.92
99	GFAP	glutamine	creatine	OCGB	82.7	0.91
100	TMEM40	VWF	leucine	OCGB	82.7	0.91
